# Sex-Specific Association of Cardiovascular Risk Factors With Migraine: The Population-Based Rotterdam Study

**DOI:** 10.1212/WNL.0000000000209700

**Published:** 2024-07-31

**Authors:** Linda Al-Hassany, Cevdet Acarsoy, M. Kamran Ikram, Daniel Bos, Antoinette MaassenVanDenBrink

**Affiliations:** From the Division of Vascular Medicine and Pharmacology, Department of Internal Medicine (L.A-H., A.M.), and Departments of Epidemiology (C.A., M.K.I., D.B.), Neurology (M.K.I.), Radiology and Nuclear Medicine (D.B.), Erasmus MC University Medical Center, Rotterdam, the Netherlands.

## Abstract

**Background and Objectives:**

Although several lines of evidence suggest a link between migraine and cardiovascular events, less is known about the association between cardiovascular risk factors (CVRFs) and migraine. This knowledge is clinically important to provide directions on mitigating the cardiovascular risk in patients with migraine. We hypothesized that CVRFs are associated with a higher migraine prevalence. Therefore, our primary objective was to investigate sex-specific associations between CVRFs and lifetime prevalence of migraine.

**Methods:**

We performed cross-sectional analyses within an ongoing population-based cohort study (Rotterdam Study), including middle-aged and elderly individuals. By means of (structured) interviews, physical examinations, and blood sampling, we obtained information on the lifetime prevalence of migraine and the following traditional CVRFs: current smoking, obesity, hypercholesterolemia, hypertension, and diabetes mellitus. Similarly, we obtained information on quantitative component data on these CVRFs, including pack-years of smoking, lipid levels, systolic and diastolic blood pressure (BP), body mass index, and fasting glucose levels. Patients with migraine were age-matched to individuals without migraine, and we performed conditional logistic regression analyses to investigate the sex-stratified association of CVRFs with migraine.

**Results:**

In total, 7,266 community-dwelling middle-aged and elderly persons were included (median age 66.6 [IQR 56.4–74.8] years, 57.5% females). The lifetime prevalence of migraine was 14.9%. In females, current smoking (odds ratio (OR) 0.72, 95% CI 0.58–0.90), more pack-years (OR per SD increase 0.91, 95% CI 0.84–1.00), diabetes mellitus (OR 0.74, 95% CI 0.56–0.98), and higher fasting glucose levels (OR per SD increase in glucose 0.90, 95% CI 0.82 − 0.98) were all related to a lower migraine prevalence while a higher diastolic BP related to a higher migraine prevalence (OR per SD increase 1.16, 95% CI 1.04–1.29). In males, no significant associations between CVRFs and migraine were observed.

**Discussion:**

Traditional CVRFs were either unrelated or inversely related to migraine in middle-aged and elderly individuals, but only in females. In males, we did not find any association between CVRFs and migraine. Because only an increased diastolic BP was related to a higher migraine prevalence in females, our study contributes to the hypothesis that migraine is not directly associated with traditional CVRFs. Future studies are warranted to extrapolate these findings to younger populations.

## Introduction

Apart from the burden due to its incapacitating headache attacks,^1,2^ migraine, particularly with aura, is increasingly linked to the occurrence of cardiovascular events, including myocardial infarction, coronary heart disease, and cardiac mortality, in males and females.^3-6^ As demonstrated in females, migraine with aura is linked to an even higher risk of major cardiovascular disease than other well-known risk factors, such as obesity and unfavorable lipid levels.^5^ One potential explanation for the association between migraine and cardiovascular disease lies in their shared underlying mechanisms because the pathophysiology of migraine is not only neuronal but also involves vascular components.^7^

In this light, it is important to acknowledge that cardiovascular risk factors may co-occur with migraine but that the link between cardiovascular risk factors in the development of migraine remains unclear. Until now, several studies^8-11^ have reported conflicting results on the association between migraine and cardiovascular risk factors. These conflicting results are probably due to differences in cardiovascular risk factors investigated, study populations, migraine assessments, and the lack of sex-stratified analyses. Indeed, differences in migraine prevalence, course, and pathophysiology between males and females contribute to the hypothesis that sex (hormones) may modify the association between cardiovascular risk factors and migraine. Yet, most of the studies deal with sex as a confounder, for example, by adjusting for sex or by using matching techniques and do not provide insights into sex differences—that is, handling sex as an effect modifier.^12^ Furthermore, no studies compared the relative contributions of these cardiovascular risk factors to the migraine prevalence. An enhanced understanding of cardiovascular risk factors involved in migraine is of clinical importance to elucidate the underlying pathophysiologic mechanisms and could ultimately lead to preventive strategies to reduce the overall cardiovascular risk in migraine.

For this study, we included categorical cardiovascular risk factors, that is, traditional risk factors that are often modifiable and included in cardiovascular risk scores, and are of direct clinical relevance as clinicians traditionally aim to tackle them. Furthermore, we also included continuous cardiovascular risk factors that are quantitative components of the cardiovascular system (e.g., instead of a categorical hypertension diagnosis, the values of systolic and diastolic blood pressure (BP)).

Therefore, the primary objective of this study was to investigate the association between the lifetime prevalence of categorical cardiovascular risk factors, including smoking status, obesity, diabetes mellitus, hypertension, and hypercholesterolemia, and migraine in a population-based cohort, taking potential sex differences into account. The secondary objective was to compare the contribution of continuous cardiovascular risk factor components, to ultimately set the stage for studies on the pathophysiology of the increased cardiovascular risk in migraine. Furthermore, we explored differences in migraine activity status.

## Methods

### Study Setting and Population

This cross-sectional study was embedded in the Rotterdam Study, a prospective population-based cohort study among middle-aged and elderly residents of Ommoord district in the city of Rotterdam in the Netherlands.^13,14^ The Rotterdam Study started in 1990 with the first cohort and subsequently extended in 2000 with the second cohort, both including participants aged 55 years or older. In 2006, the study expanded once again with the third cohort, including individuals aged 45–54 years as well.

### Assessment of Migraine Diagnoses

Lifetime migraine diagnoses were assessed through a structured migraine questionnaire that was administered during a face-to-face (home) or phone interview (n = 634, because of logistic reasons) by trained interviewers. Diagnostic criteria were based on the second edition of the International Classification of Headache Disorders (ICHD-II),^15^ and items were modified from a validated questionnaire used in the Genetic Epidemiology of Migraine study of Leiden, the Netherlands.^16^

As described previously,^17^ only a positive answer to the screening question of whether the participant ever experienced a headache with severe pain intensity that affected their daily activities led to a continuation of the remainder of the migraine questionnaire. If either a negative answer or other causes (e.g., tumor or stroke) for the severe headache were provided, no further headache-related questions were asked. Moreover, migraine with aura was defined as meeting all criteria for migraine without aura (i.e., five headache attacks instead of 2) accompanied by aura symptoms lasting between 5 and 60 minutes.

For this study, we only included participants of whom the migraine assessment (i.e., having a history of migraine, active migraine, or no migraine) derived from the 3 cohorts within the Rotterdam Study was available. Subjects who were classified as “probable migraine” according to the ICHD criteria^15^ (fulfilling all except for one of the migraine criteria) were regarded as individuals without migraine. To explore differences in migraine activity status, individuals with migraine were further dichotomized into having a history of migraine (>1 year since the last attack) or active migraine (<1 year since the last attack).

### Assessment of Cardiovascular Risk Factors and Confounding Variables

Data on smoking status (never, former, or current smoker), alcohol consumption (grams/day), physical activity by quantifying activity intensity (metabolic equivalent of a task in hours per week),^18^ educational level (categorized), and antihypertensive as well as lipid-lowering drug use were self-reported and collected through interview. Pack-years was calculated as the number of cigarettes smoked per day multiplied by the number of years smoked, divided by 20.

Cardiometabolic risk factors and anthropometric variables were collected by physical examination and blood sampling during a visit to the research center. Serum glucose (in a fasting state), total cholesterol and high-density lipoprotein cholesterol (HDL-cholesterol), and triglyceride levels (all expressed in mmol/L) were measured using standard laboratory techniques at the Erasmus Medical Center.^14^

BP measurements were performed at the right brachial artery with a random-zero sphygmomanometer in a sitting position. The average of 2 BP measurements with a two-minute interval was used. Hypertension was defined based on the use of antihypertensive medication and/or an average systolic ≥140 mm Hg or diastolic ≥90 mm Hg of 2 BP measurements. Hypercholesterolemia was identified as total cholesterol of ≥6.2 mmol/L or the use of lipid-lowering agents. Diabetes mellitus was diagnosed on the basis of a fasting plasma glucose level of ≥7.0 mmol/L or antidiabetic medication use. Obesity was defined as a body mass index (BMI) >30 kg/m^2^.

### Statistical Analyses

To analyze the association between categorical and continuous cardiovascular risk factors and migraine prevalence, we used the following strategy. First, missing variables were imputed as described below. Second, individuals with migraine were age-matched to individuals without migraine (ratio 1:3) to adjust for the potential confounding effect of age. Third, we performed univariable and multivariable conditional logistic regression analyses to investigate the association of categorical cardiovascular risk factors with migraine, stratified by sex, using 3 models. In the first model, we studied the unadjusted association between the individual categorical cardiovascular risk factors and the prevalence of lifetime migraine. The second model was constructed to study the association of the separate categorical cardiovascular risk factors with migraine, while adjusting for potential confounders: alcohol consumption, physical activity, educational level, and study cohort. The third model contained all categorical cardiovascular risk factors and confounding variables simultaneously.

We applied an approach similar to that mentioned above to investigate the association of continuous cardiovascular risk factor components with migraine. One difference was that the continuous cardiovascular risk factor components in the second and third models were standardized and expressed per SD, for females and males separately, to allow a direct and intuitive comparison of their individual contribution.

Given the previously described notable effect of migraine activity status^19,20^—that is, the distinction between having a history of migraine vs active migraine—on cardiovascular risk among patients with migraine, we conducted exploratory analyses to examine the effects of migraine activity status. In alignment with the approach used for our primary objective, we employed 3 models to analyze both categorical and continuous cardiovascular risk factors. For these exploratory analyses, however, we applied multinomial logistic regression models allowing the dependent variable to have more than 2 categories. The status of never having reported migraine served as the reference category. Given the small sample sizes, we adjusted for age rather than using an age-matching strategy. All other confounders were kept the same as in our primary analyses.

Odds ratios (ORs) with the 95% CI were calculated by applying the conditional logistic regression analyses and multinomial logistic regression models on both research questions. Data of most variables (i.e., smoking status, alcohol consumption, use of antihypertensives/lipid-lowering drugs/antidiabetics, educational level, BMI, systolic and diastolic BP, total cholesterol, glucose, HDL-cholesterol, waist-to-hip ratio, and triglycerides) were missing for less than 10% of participants, except for physical activity (18.5%) (Table 1). Missing values were imputed using multiple imputation, with 10 imputations and 10 iterations using the “mice” package in R. We used IBM SPSS (Chicago, IL) version 27 and R version 4.0.5 for Windows to perform all analyses.

**Table 1:** Baseline Characteristics of the Total Rotterdam Study Population, Including Individuals With and Without Migraine

Total study population	All (n = 7,266)	With (a history of) migraine (n = 1,085)	Without (a history of) migraine (n = 6,181)
Aura (%)	221 (3.0)	221 (20.4)	—
Female (%)	4,181 (57.5)	884 (81.5)	3,297 (53.3)
Age (y)	66.6 [56.4−74.8]	62.2 [55.5−72.8]	66.9 [56.5−75.2]
Education level (%)			
Primary education	692 (9.5)	100 (9.2)	592 (9.6)
Lower/intermediate general education or lower vocational education	2,906 (40.0)	490 (45.2)	2,416 (39.1)
Intermediate vocational education or higher general education	2,114 (29.1)	273 (25.2)	1841 (29.8)
Higher vocational education or university	1,554 (21.4)	222 (20.5)	1,332 (21.5)
BMI (kg/m^2^)	27.1 [24.7−29.9]	27.3 [24.5−30.1]	27.0 [24.7−29.9]
Obesity (BMI >30 kg/m^2^) (%)	1764 (24.3)	276 (25.4)	1,488 (24.1)
Waist-to-hip ratio	0.88 [0.82−0.95]	0.86 [0.80−0.91]	0.89 [0.83−0.95]
Systolic blood pressure (mm Hg)	139 [125−155]	138 [124−154]	140 [126−156]
Diastolic blood pressure (mm Hg)	83 [76−91]	83 [77−91]	83 [76−91]
Hypertension (%)	4,963 (68.3)	720 (66.4)	4,243 (68.6)
Antihypertensive drug use (%)	2,960 (40.7)	435 (40.1)	2,525 (40.9)
Total cholesterol (mmol/L)	5.4 [4.8−6.2]	5.6 [4.9−6.3]	5.4 [4.7−6.1]
High-density lipoprotein cholesterol (mmol/L)	1.4 [1.2−1.7]	1.5 [1.2−1.8]	1.4 [1.1−1.7]
Triglycerides (mmol/L)	1.3 [1.0−1.7]	1.3 [1.0−1.8]	1.3 [1.0−1.7]
Hypercholesterolemia (%)	3,590 (49.4)	556 (51.2)	3,034 (49.1)
Lipid-lowering medication use (%)	1979 (27.2)	288 (26.5)	1,691 (27.4)
Fasting glucose levels (mmol/L)	5.4 [5.0−5.9]	5.3 [5.0−5.8]	5.4 [5.1−5.9]
Diabetes mellitus (%)	857 (11.8)	101 (9.3)	756 (12.2)
Smoking status (%)			
Former	3,601 (49.6)	532 (49.0)	3,069 (49.7)
Current	1,277 (17.6)	159 (14.7)	1,118 (18.1)
Pack-years of ever smokers	16.8 [6.0−33.0]	12.5 [3.5−28.7]	17.5 [6.3−33.8]
Alcohol consumption (gram/day)	6.4 [0.5−8.6]	3.8 [0.5−8.6]	6.4 [0.5−8.6]
Physical activity (MET hours/week)	37.3 [15.0−75.6]	39.2 [15.2−74.5]	37.1 [15.0−75.7]

Abbreviations: BMI = body mass index; MET = metabolic equivalent of task.

Note: Imputed and unadjusted values of the cohort, with and without stratification for the migraine diagnosis, are displayed as means (SD) or medians [interquartile range, Q_3_–Q_1_] or as numbers (percentage). Anderson-Darling normality test was used to assess normality.

Missing values of variables before imputation: smoking status: 0.04%; alcohol consumption: 0.22%; antihypertensive drug use: 0.45%; lipid-lowering drug use: 0.45%; antidiabetic drug use: 0.45%; educational level: 0.88%; BMI: 6.76%; systolic BP: 8.02%; diastolic BP: 8.02%; total cholesterol: 8.55%; glucose: 8.56%; high-density lipoprotein cholesterol: 8.75%; waist-to-hip ratio: 9.01%; triglycerides: 9.47%; and physical activity: 18.54%.

### Standard Protocol Approvals, Registrations, and Patient Consents

The Medical Ethics Committee of the Erasmus Medical Center (Erasmus MC; registration number MEC 02.1015) and the Dutch Ministry of Health, Welfare, and Sport (Population Screening Act WBO, license number 1071272-159521-PG) have approved the Rotterdam Study. The Rotterdam Study Personal Registration Data collection is filed with the Erasmus MC Data Protection Officer under registration number EMC1712001. The Rotterdam Study has been entered into the Netherlands National Trial Register and the WHO International Clinical Trials Registry Platform under shared catalog number NTR6831. All participants provided written informed consent to participate in the study and to have their information obtained from treating physicians.

### Data Availability

Data can be obtained upon request. Requests should be directed toward the management team of the Rotterdam Study (secretariat.epi@erasmusmc.nl), which has a protocol for approving data requests. Because of restrictions based on privacy regulations and informed consent of the participants, data cannot be made freely available in a public repository.

## Results

In this study, a total of 7,266 participants, for whom migraine assessment data were available, were included. Baseline characteristics of the total cohort and stratified for migraine diagnosis are presented in Table 1, showing that a total of 1,085 participants (14.9%) fulfilled the criteria for active migraine (n = 422, 38.9%) or a history of migraine (n = 663, 61.1%), and 306 participants (4.2%) were classified as probable migraine. Among individuals with migraine, 221 (20.4%) suffered from aura symptoms. Individuals with migraine were younger (62.2 [55.5–72.8] years) than individuals without migraine (66.9 [56.5–75.2] years). Furthermore, there was a difference in migraine prevalence between the sexes; among the participants, 201 of 3,085 males (6.5%) reported having active migraine or a history of migraine, compared with 884 of 4,181 females (21.1%). Tables 2 and 3 show baseline characteristics for males and females.

**Table 2:** Baseline Characteristics of Male Rotterdam Study Participants, Including Individuals With and Without Migraine

Male participants	All (n = 3,085)	With (a history of) migraine (n = 201)	Without (a history of) migraine (n = 2,884)
Aura (%)	37 (1.2)	37 (18.4)	—
Age (y)	66.2 [56.2−74.5]	61.4 [55.8−72.5]	66.5 [56.2−74.6]
Education level (%)			
Primary education	237 (7.7)	20 (10.0)	217 (7.5)
Lower/intermediate general education or lower vocational education	809 (26.2)	58 (28.9)	751 (26.0)
Intermediate vocational education or higher general education	1,125 (36.5)	60 (29.9)	1,065 (36.9)
Higher vocational education or university	914 (29.6)	63 (31.3)	851 (29.5)
BMI (kg/m^2^)	27.2 [25.0−29.6]	27.5 (3.3)	27.1 [25.0−29.6]
Obesity (BMI >30 kg/m^2^)	672 (21.8)	40 (19.9)	632 (21.9)
Waist-to-hip ratio	0.94 [0.89−0.99]	0.94 (0.08)	0.94 [0.89−0.99]
Systolic blood pressure (mm Hg)	141 [127−156]	140 [127−154]	141 [127.8−156]
Diastolic blood pressure (mm Hg)	84 [77−91]	83 [76−90]	84 [77−91]
Hypertension (%)	2,180 (70.7)	141 (70.1)	2039 (70.7)
Antihypertensive drug use (%)	1,305 (42.3)	94 (46.8)	1,211 (42.0)
Total cholesterol (mmol/L)	5.2 [4.5−5.9]	5.2 (1.0)	5.2 [4.5−5.9]
High-density lipoprotein cholesterol (mmol/L)	1.3 [1.0−1.5]	1.3 [1.0−1.5]	1.3 [1.0−1.5]
Triglycerides (mmol/L)	1.3 [1.0−1.8]	1.4 [1.1−1.9]	1.3 [1.0−1.8]
Hypercholesterolemia (%)	1,457 (47.2)	97 (48.3)	1,360 (47.2)
Lipid-lowering medication use (%)	989 (32.1)	68 (33.8)	921 (31.9)
Fasting glucose levels (mmol/L)	5.5 [5.2−6.1]	5.5 [5.2−6.2]	5.5 [5.2−6.1]
Diabetes mellitus (%)	439 (14.2)	30 (14.9)	409 (14.2)
Smoking status (%)			
Former	1819 (59.0)	129 (64.2)	1,690 (58.6)
Current	533 (17.3)	30 (14.9)	503 (17.4)
Pack-years of ever smokers	20.0 [8.0−36.8]	20.0 [8.2−34.9]	20.0 [8.0−37.0]
Alcohol consumption (gram/day)	8.6 [1.6−20.0]	6.4 [1.6−10.7]	8.6 [1.6−20.0]
Physical activity (MET hours/week)	34.8 [15.0−68.7]	37.3 [18.0−70.3]	34.5 [14.8−68.5]

Abbreviations: BMI = body mass index; MET = metabolic equivalent of task.

Note: Imputed and unadjusted values of the cohort, with and without stratification for the migraine diagnosis, are displayed as means (SD) or medians [interquartile range, Q_3_–Q_1_] or as numbers (percentage). Shapiro-Wilk normality test was used to assess normality.

**Table 3:** Baseline Characteristics of Female Rotterdam Study Participants, Including Individuals With and Without Migraine

Female participants	All (n = 4,181)	With (a history of) migraine (n = 884)	Without (a history of) migraine (n = 3,297)
Aura (%)	184 (4.4)	184 (20.8)	—
Age (y)	66.8 [56.4−75.1]	62.6 [55.4−72.8]	67.2 [56.7−75.7]
Education level (%)			
Primary education	455 (10.9)	80 (9.0)	375 (11.4)
Lower/intermediate general education or lower vocational education	2097 (50.2)	432 (48.9)	1,665 (50.5)
Intermediate vocational education or higher general education	989 (23.7)	213 (24.1)	776 (23.5)
Higher vocational education or university	640 (15.3)	159 (18.0)	481 (14.6)
BMI (kg/m^2^)	27.0 [24.3−30.2]	27.2 [24.2−30.3]	26.9 [24.3−30.2]
Obesity (BMI >30 kg/m^2^)	1,092 (26.1)	236 (26.7)	856 (26.0)
Waist-to-hip ratio	0.84 [0.79−0.89]	0.84 [0.79−0.89]	0.84 [0.80−0.90]
Systolic blood pressure (mm Hg)	138 [123−155]	137 [123−154]	138 [124−156]
Diastolic blood pressure (mm Hg)	83 [76−91]	83 [77−92]	83 [76−91]
Hypertension (%)	2,783 (66.6)	579 (65.5)	2,204 (66.8)
Antihypertensive drug use (%)	1,655 (39.6)	341 (38.6)	1,314 (39.9)
Total cholesterol (mmol/L)	5.6 [5.0−6.3]	5.6 [5.0−6.3]	5.6 [5.0−6.3]
High-density lipoprotein cholesterol (mmol/L)	1.5 [1.3−1.8]	1.5 [1.3−1.8]	1.5 [1.3−1.9]
Triglycerides (mmol/L)	1.2 [0.9−1.7]	1.3 [0.9−1.7]	1.2 [0.9−1.7]
Hypercholesterolemia (%)	2,133 (51.0)	459 (51.9)	1,674 (50.8)
Lipid-lowering medication use (%)	990 (23.7)	220 (24.9)	770 (23.4)
Fasting glucose levels (mmol/L)	5.3 [5.0−5.8]	5.3 [4.9−5.7]	5.3 [5.0−5.8]
Diabetes mellitus (%)	418 (10.0)	71 (8.0)	347 (10.5)
Smoking status (%)			
Former	1782 (42.6)	403 (45.6)	1,379 (41.8)
Current	744 (17.8)	129 (14.6)	615 (18.7)
Pack-years of ever smokers	13.8 [4.0−30.0]	9.9 [2.5−26.8]	15.0 [4.5−30.3]
Alcohol consumption (gram/day)	2.0 [0.5−8.6]	1.6 [0.5−8.6]	3.8 [0.5−8.6]
Physical activity (MET hours/week)	40.1 [15.0−81.2]	39.5 [15.0−75.3]	40.5 [15.0−82.1]

Abbreviations: BMI = body mass index; MET = metabolic equivalent of task.

Note: Imputed and unadjusted values of the cohort, with and without stratification for the migraine diagnosis, are displayed as means (SD) or medians [interquartile range, Q_3_–Q_1_] or as numbers (percentage). Shapiro-Wilk normality test was used to assess normality.

A strong cohort effect was observed in the median age and distribution of cardiovascular risk factors, despite age-matching, in individuals with and without migraine (median age in cohort 1: 78.6 [75.5–82.5] years; in cohort 2: 71.3 [68.8–74.5] years; in cohort 3: 56.9 [52.3–60.6] years) (see eTables 1–3). Therefore, we additionally adjusted for this cohort effect.

### Categorical Cardiovascular Risk Factors

Current smoking (OR 0.72, 95% CI 0.58–0.89) and diabetes mellitus (OR 0.75, 95% CI 0.57–0.99) were statistically significantly associated with a lower migraine prevalence in females only (see Table 4). The third model also confirmed that this association was approximately as strong for current smoking (OR 0.72, 95% CI 0.58–0.90) as for diabetes mellitus (OR 0.74, 95% CI 0.56–0.98) in females only.

**Table 4:** Associations of Categorical Cardiovascular Risk Factors With Migraine, Stratified by Sex

Cardiovascular risk factor	Model 1		Model 2		Model 3	
	Female participants	Male participants	Female participants	Male participants	Female participants	Male participants
Current smoking	0.68 [0.55−0.83]	0.78 [0.50−1.21]	0.72 [0.58−0.89]	0.82 [0.52−1.29]	0.72 [0.58−0.90]	0.84 [0.53−1.33]
Obesity	1.04 [0.87−1.24]	0.83 [0.56−1.23]	1.01 [0.84−1.20]	0.86 [0.58−1.29]	1.02 [0.85−1.22]	0.79 [0.51−1.20]
Hypercholesterolemia	1.07 [0.92−1.25]	0.99 [0.72−1.37]	1.09 [0.93−1.28]	1.02 [0.73−1.43]	1.11 [0.95−1.30]	0.99 [0.70−1.39]
Hypertension	1.08 [0.91−1.28]	1.18 [0.82−1.72]	1.08 [0.90−1.28]	1.20 [0.82−1.75]	1.08 [0.90−1.29]	1.24 [0.83−1.84]
Diabetes Mellitus	0.79 [0.60−1.03]	1.21 [0.77−1.90]	0.75 [0.57−0.99]	1.17 [0.74−1.85]	0.74 [0.56−0.98]	1.23 [0.76−1.99]

Model 1 is unadjusted.

Model 2 is adjusted for Rotterdam Study cohort, alcohol consumption, physical activity, and educational level.

Model 3 is adjusted for current smoking, obesity, hypercholesterolemia, hypertension, diabetes mellitus, Rotterdam Study cohort, alcohol consumption, physical activity, and educational level.

### Continuous Cardiovascular Risk Factor Components

Results on individual components of the cardiovascular system are presented in Table 5. The second model indicates that an adjusted increase in pack-years was associated with a slightly lower prevalence of migraine (OR per SD increase 0.92, 95% CI 0.84–1.00) in females. In addition, a higher diastolic BP was associated with a slightly higher prevalence of migraine (OR per SD increase 1.13, 95% CI 1.04–1.22) in females. In the multivariable adjusted third model, the effect estimates of diastolic BP were the highest (OR per SD increase 1.16, 95% CI 1.04–1.29), followed by fasting glucose levels (OR per SD increase 0.90, 95% CI 0.82–0.98) and finally by pack-years (OR per SD increase 0.91, 95% CI 0.84–1.00). These associations were only observed in females, and none of the cardiovascular risk factors were significantly associated with migraine in males.

**Table 5:** Association of Continuous Cardiovascular Determinants and Migraine, Stratified by Sex

Cardiovascular Risk factor	Model 1		Model 2		Model 3	
	Female participants	Male particpants	Female participants	Male particpants	Female participants	Male particpants
Pack-years	0.99 [0.99−1.00]	1.00 [0.99−1.01]	0.92 [0.84−1.00]	1.03 [0.87−1.22]	0.91 [0.84−1.00]	1.04 [0.87−1.24]
Total cholesterol^a^	0.99 [0.92−1.07]	0.99 [0.85−1.16]	1.00 [0.92−1.08]	1.05 [0.89−1.25]	0.97 [0.89−1.05]	1.04 [0.87−1.25]
HDL-cholesterol^a^	0.84 [0.70−1.01]	1.26 [0.80−1.98]	0.95 [0.88−1.03]	1.15 [0.97−1.36]	0.96 [0.88−1.06]	1.16 [0.95−1.41]
Triglycerides^a^	1.08 [0.97−1.20]	0.94 [0.80−1.10]	1.05 [0.97−1.13]	0.96 [0.80−1.15]	1.06 [0.96−1.16]	1.01 [0.82−1.23]
Systolic blood pressure^b^	1.00 [1.00−1.01]	1.00 [0.99−1.00]	1.06 [0.97−1.16]	0.96 [0.80−1.14]	0.95 [0.84−1.07]	0.98 [0.77−1.26]
Diastolic blood pressure^b^	1.01 [1.00−1.02]	0.99 [0.98−1.01]	1.13 [1.04−1.22]	0.95 [0.80−1.12]	1.16 [1.04−1.29]	0.96 [0.76−1.22]
BMI^c^	1.01 [0.99−1.02]	0.98 [0.94−1.03]	1.03 [0.95−1.11]	0.95 [0.81−1.13]	1.01 [0.93−1.10]	0.99 [0.83−1.19]
Fasting glucose levels^a^	0.94 [0.87−1.01]	1.02 [0.90−1.15]	0.92 [0.85−1.00]	1.04 [0.88−1.22]	0.90 [0.82−0.98]	1.06 [0.89−1.25]

Model 1 is unadjusted.

Model 2 is adjusted for Rotterdam Study cohort, alcohol consumption, physical activity, and educational level. All continuous risk factors have been expressed as a Z-score for females and males separately.

Model 3 is adjusted for former smoking, current smoking, total cholesterol, HDL-cholesterol, triglycerides, systolic BP, diastolic BP, BMI, fasting glucose levels, Rotterdam Study cohort, alcohol consumption, physical activity, and educational level. All continuous risk factors have been expressed as a Z-score for females and males separately.

a Expressed in mmol/L.

b Expressed in mm Hg.

c Expressed in kg/m^2^.

### Exploratory Analyses on Migraine Activity Status

Our cohort included in total 351 females (39.7%) and 71 males (35.3%) with active migraine.

As shown in eTable 4, the adjusted odds of the probability of having a history of migraine (vs never migraine) was consistently higher among females with hypercholesterolemia (OR 1.24, 95% CI 1.03–1.50 in model 3) or hypertension (OR 1.34, 95% CI 1.06–1.70 in model 3). The odds of the probability of having active migraine (vs never migraine) among females with these risk factors were substantially lower yet nonsignificant. Notably, current smoking emerged as the only cardiovascular risk factor significantly associated with lower odds of both having a history of migraine (OR 0.73, 95% CI 0.55–0.96 in model 3) or active migraine (OR 0.69, 95% CI 0.51–0.94 in model 3). For males, no significant associations were observed for either migraine history or active migraine in multivariable adjusted models.

A similar pattern was observed for several continuous cardiovascular risk factors, as presented in eTable 5, showing a higher adjusted odds of the probability of having a history of migraine (vs never migraine) among females for higher diastolic blood pressure (OR per SD increase 1.17, 95% CI 1.04–1.33 in model 3). The odds of the probability of having active migraine (vs never migraine) among females with an OR per SD increase of the continuous cardiovascular risk factors were overall lower yet nonsignificant. Furthermore, in general, ORs of cardiovascular risk factors in males having a history of migraine were higher compared with males having active migraine (vs never migraine), although none of these associations were significant.

## Discussion

In our middle-aged and elderly cohort of participants from the general population, of which 1,085 were diagnosed with migraine, we did not observe a higher burden of traditional cardiovascular risk factors such as obesity, hypercholesterolemia, or hypertension compared with individuals without migraine, while smoking and diabetes mellitus were associated with a lower migraine prevalence in females. By contrast, a higher diastolic BP was associated with a higher migraine prevalence in females.

Hitherto, clinical and epidemiologic studies are often subject to clinical and methodological heterogeneity, which might be caused by (1) different age ranges and the lack of sex-specific analyses, (2) information (recall) bias due to diverse assessments of cardiovascular risk factors and migraine, (3) selection bias due to clinic- or hospital-based vs population-based cohorts, and (4) confounding bias due to unidentified or unadjusted confounders related to cardiovascular health or lifestyle.^21,22^ In this sense, our study provides additional insights into previous analyses that, compared with our cohort, focused on a younger population with a low cardiovascular risk.^23^

We found no associations between migraine and different lipid levels (or hypercholesterolemia) or obesity, which does not confirm earlier studies on this topic.^21,24,25^ This might be explained by our older cohort, as an association with obesity was mainly described in individuals of reproductive age.^26^

Smoking may trigger migraine attacks^27^ because cigarette smoke may act as an agonist of transient receptor potential ankyrin 1 (TRPA1) channels,^28^ which upon activation mediate release of calcitonin gene-related peptide (CGRP)—a key molecule in migraine pathophysiology.^29^ As our cohort is substantially older than other study populations,^30^ we hypothesize that the association of current smoking and a lower migraine prevalence stems from lifestyle (behavioral) choices. Although a similar pattern would also be expected in males, this sex difference might, apart from the obviously lower statistical power due to the lower prevalence of migraine in males, be related to the differential regulation of TRPA1 channels in males and females.^29^ Alternatively, gender-related and social factors or cultural norms might also serve as an explanation for the noted disparities, such as the societal acceptance of smoking among elderly men vs women, and the potentially greater acceptance for women to abstain from smoking because of migraine compared with men.

Our findings are in line with a previous case-control study supporting an inverse association between diabetes and migraine^31^ and with a cohort study including only females in a similar age range as ours.^32^ (Reactive) hypoglycemia has been regarded as a trigger of migraine^33^ and might explain the increased plasma glucose levels and decreased migraine prevalence. In addition, CGRP—as mentioned above causally linked to migraine^34^—has a suppressive role on insulin release.^35^ Another hypothesis is that developing diabetes could lead to impairment of sensory nerves, with a subsequent reduction of the vasodilatory and nociceptive effects of CGRP and a lower migraine prevalence.^36,37^ We only found an inverse association between diabetes mellitus and migraine in females and, although we assume that most of the females in our study were postmenopausal (average age of 67 years), sex hormones might potentially be involved in this study.^38^

Our observation of a positive association between increased diastolic BP and migraine is in line with previous epidemiologic findings^39^ and genome-wide cross-phenotype meta-analysis,^40^ hinting toward a crucial role of diastolic BP in migraine susceptibility. While our effect estimates were marginal, the absence of an association with systolic BP (and hypertension) is reflected by the essential difference between the systolic BP—a measure of pressure against (macro)vascular arteries—and diastolic BP—a measure of (micro)vascular and peripheral resistance. Therefore, the increased diastolic BP in patients with migraine might indicate (slightly) reduced microvascular function, the same system where CGRP mainly exerts its effects.^41^ While the diastolic and systolic BP cannot be evaluated in isolation^42^ and lower systolic BP was reported in a previous study,^39^ the absence of such effects in our study might be explained by the relatively old individuals who already suffer from atherosclerosis and hypertension because of aging. The absence of an association between hypertension and an increased migraine prevalence in males notably contradicts previous age-adjusted findings from the Physicians' Health Study, which indicated that apparently healthy and marginally younger males (with a mean age of approximately 58 years) more frequently reported hypertension.^43^ However, no obvious differences were observed for systolic and diastolic BP as well as BMI.^43^

Furthermore, we explored the association with cardiovascular risk factors in individuals with a history of migraine or active migraine with those who never had migraine. Our findings indicated that, in general, females with traditional cardiovascular risk factors are more likely to have a history of migraine rather than active migraine, compared with those who have never experienced migraine, independent of age, confirming results from the Women's Health Study including women aged 45 years and older at baseline.^20^ While caution is absolutely necessary in drawing causal conclusions from these results, they could support the hypothesis that active migraine is associated with a more healthy vascular status.^20^

In addition, these analyses revealed that in individuals with a history of migraine, compared with individuals who never had migraine, the distinct effect of the diastolic BP and its differences with systolic BP diminish—likely because of a combination of the aforementioned microvascular and macrovascular deterioration.

Compared with individuals without migraine, patients with migraine seem to have less arterial calcifications,^44^ although arterial stiffness has been recognized as a pivotal cardiovascular risk factor.^45^ These findings contribute to the hypothesis that the increased cardiovascular risk observed in migraine might be associated with alternative pathways, rather than to traditional cardiovascular risk factors that lead to impaired peripheral endothelial function and arterial stiffness.^44,46^ Indeed, CGRP might have (vaso)protective and preventive effects against atherosclerosis.^34^ These effects may (partly) be mediated by transient receptor potential vanilloid 1 channel activation, of which the activation and/or expression is modulated by (sex) steroids^29^—despite the absence of evident sex differences in this study. Alternative mechanisms that have been hypothesized to provide a link between migraine and cardiovascular risk factors include, for example, hypercoagulability and inflammation.^10,47^ Nevertheless, it is crucial to acknowledge that this population-based cohort study does not permit definitive mechanistic conclusions, although it may lay the groundwork for hypotheses on the pathophysiology in future basic and translational research.

A major strength of our study is that cardiovascular risk factors and migraine were assessed in a standardized manner within both sexes in a population-based setting. Indeed, we used a validated interview to ascertain the migraine diagnoses in a population-based cohort, allowing generalizability to the overall (Dutch) population.

A limitation of our study is that we cannot exclude that the smaller power in males, because of the much lower prevalence of migraine compared with that in females, might underlie the absence of any associations between migraine and cardiovascular risk factors in males. Another potential explanation for the lack of differences between males with and without migraine in our primary analyses could be that, compared with females, males with migraine are reported to have less severe attacks and lower levels of disability.^48^ Therefore, this group may have been misclassified as having “probable migraine,” a group which we considered as “no migraine” in our study. In addition, we advise caution when drawing any causal conclusions from these cross-sectionally obtained effect estimates of the third model. While several continuous measurements (e.g., BP, blood glucose, and BMI) were measured during a single research visit, they do not capture the duration or temporal aspect of most categorical risk factors, especially in relation to the migraine onset or diagnosis. Moreover, they cannot be directly compared with or account for long-standing cardiovascular risk factors such as hypertension or diabetes mellitus.

Furthermore, working in the domain of causal inference, we advise caution in the direct interpretation of these (Z-transformed) ORs, considering that adjusting for all cardiovascular and confounding risk factors at once may “open” other pathways, leading to collider bias. Yet, comparing the effect estimates of the first and third model, no striking differences were observed and all effect estimates remained in the same direction without changing the confidence intervals materially. In addition, participants who have (had) moderately (but not severely) painful headache attacks and/or aura symptoms without headache could be misclassified as false-negative. Furthermore, individuals experiencing migraine with aura may have been misclassified as individuals with migraine without aura or even no migraine at all, because of the first criterion requiring at least 5 headache attacks—leading to underestimation of our results. Using the ICHD-2 criteria instead of the more current ICHD-3 guidelines, which were not yet available at the time data were collected within the Rotterdam Study, may have resulted in subtle deviations in migraine diagnoses.

Although we conducted exploratory analyses to examine the independent effect of aura symptoms on the relationship between migraine and cardiovascular risk factors in both sexes (*data not shown*), these analyses revealed no profound differences between persons with migraine with or without aura. However, we acknowledge that our study did not specifically focus on the independent effects of aura symptoms, and the lack of observed differences could potentially be attributed to insufficient statistical power. Finally, we could not differentiate between type I and type II diabetes and had no data on insulin levels. Therefore, hypotheses of impaired insulin sensitivity in migraine could not be refuted nor confirmed.^49^

Further translational studies are needed to test our proposed hypothesis that the association between migraine and cardiovascular disease cannot be explained by traditional cardiovascular risk factors, as previously demonstrated,^10^ but are rather mediated by underexposed factors representing microvascular dysfunction.^47^ These studies should additionally consider additional cardiovascular risk factors that were not available in this study, such as microemboli, patent foramen ovale, vasospasm, fibromuscular dysplasia, arterial dissection, and atrial fibrillation.

Studies with larger sample sizes are also warranted to incorporate lifetime changes in migraine and study similar associations with cardiovascular components in younger subjects. The latter aspect is of special importance, considering that age plays a major role in the deterioration of the cardiovascular system,^50^ and CGRP levels and migraine prevalence have been described to decrease with age.^2,34^ Moreover, the association between migraine and cardiovascular risk and disease might differ between different age categories. Indeed, a previous study reports an opposite pattern of association between migraine with aura and ischemic stroke—present in women with the lowest cardiovascular risk, including younger individuals—and migraine with aura and myocardial infarction—present only in women with the highest cardiovascular risk, including elderly.^47^ In addition, at the ages in this cohort, most cardiovascular risk factors are common, perhaps masking the duration of cardiovascular risk factors. Previous cohort studies, such as the Nurses' Health Study, examined the long-term risks of migraine and have noted a higher prevalence of risk factors for individuals at rather younger ages (range 25–42 years).^6^

In conclusion, despite the established link between migraine and cardiovascular events, in our current study, most traditional cardiovascular risk factors are not associated with an increased migraine prevalence in middle-aged and elderly participants. We did find in females, that diabetes mellitus, higher glucose levels, and smoking are associated with a decreased migraine prevalence while a higher diastolic BP is associated with an increased prevalence of migraine. Although definitive conclusions about underlying biological mechanisms in migraine cannot be drawn, these findings might support involvement of nontraditional factors and the presence of impaired microvascular function, reflected by increased diastolic BP.

## Appendix Authors

**Table:**

Name	Location	Contribution
Linda Al-Hassany, MSc	Division of Vascular Medicine and Pharmacology, Department of Internal Medicine, Erasmus MC University Medical Center, Rotterdam, the Netherlands	Drafting/revision of the manuscript for content, including medical writing for content; study concept or design; analysis or interpretation of data
Cevdet Acarsoy, MSc	Department of Epidemiology, Erasmus MC University Medical Center, Rotterdam, the Netherlands	Revision of the manuscript for content, including medical writing for content; role in the acquisition of data; analysis or interpretation of data
M. Kamran Ikram, MD, PhD	Department of Epidemiology; Department of Neurology, Erasmus MC University Medical Center, Rotterdam, the Netherlands	Revision of the manuscript for content, including medical writing for content; study concept or design; analysis or interpretation of data
Daniel Bos, MD, PhD	Department of Epidemiology; Department of Radiology and Nuclear Medicine, Erasmus MC University Medical Center, Rotterdam, the Netherlands	Revision of the manuscript for content, including medical writing for content; study concept or design; analysis or interpretation of data
Antoinette MaassenVanDenBrink, PhD	Division of Vascular Medicine and Pharmacology, Department of Internal Medicine, Erasmus MC University Medical Center, Rotterdam, the Netherlands	Revision of the manuscript for content, including medical writing for content; study concept or design; analysis or interpretation of data

## Glossary

BMI: body mass index
BP: blood pressure
CGRP: Calcitonin Gene-Related Peptide
CVRFs: cardiovascular risk factors
HDL: high-density lipoprotein
OR: odds ratios
TRPA1: transient receptor potential ankyrin 1
